# Impact Resistance of Epoxy Composites Reinforced with Amazon Guaruman Fiber: A Brief Report

**DOI:** 10.3390/polym13142264

**Published:** 2021-07-10

**Authors:** Raphael H. M. Reis, Fabio C. Garcia Filho, Larissa F. Nunes, Veronica S. Candido, Alisson C. R. Silva, Sergio N. Monteiro

**Affiliations:** 1 Department of Materials Science, Military Institute of Engineering, Rio de Janeiro 22290-270, RJ, Brazil; raphaelreis@ime.eb.br (R.H.M.R.); larissafernandes@ime.eb.br (L.F.N.); sergio.neves@ime.eb.br (S.N.M.); 2 Department of Mechanical and Aerospace Engineering, University of California San Diego—UCSD, La Jolla, CA 92093, USA; 3 Engineering of Natural Resources of the Amazon Program, Federal University of Para, Ananindeua 67030-007, PA, Brazil; scarpini@ufpa.br (V.S.C.); alissonrios@ufpa.br (A.C.R.S.)

**Keywords:** guaruman fiber, epoxy composite, Izod impact test

## Abstract

Fibers extracted from Amazonian plants that have traditionally been used by local communities to produce simple items such as ropes, nets, and rugs, are now recognized as promising composite reinforcements. This is the case for guaruman (*Ischinosiphon körn*) fiber, which was recently found to present potential mechanical and ballistic properties as 30 vol% reinforcement of epoxy composites. To complement these properties, Izod impact tests are now communicated in this brief report for similar composites with up to 30 vol% of guaruman fibers. A substantial increase in impact resistance, with over than 20 times the absorbed energy for the 30 vol% guaruman fiber composite, was obtained in comparison to neat epoxy. These results were statistically validated by Weibull analysis, ANOVA, and Tukey’s test. Scanning electron microscopy analysis disclosed the mechanisms responsible for the impact performance of the guaruman fiber composites.

## 1. Introduction

Since the beginning of the 21st century, topics such as recyclability, renewability, and biodegradability have become mandatory, and strongly influence the processing and development of novel materials [1,2,3]. In particular, a new trend for designing “greener materials” made of natural lignocellulosic fibers (NLFs) is revealing potential materials for the reinforcement of composites for engineering applications [4,5,6,7,8]. Indeed, several NLF-reinforced polymer matrix composites exhibit promising results in different industries, such as the automobile [9,10], aerospace [11], packing [12], and building [13,14,15] industries, as well as, more recently, even as impact-resistant materials for use against high-energy ammunition [16,17,18,19,20].

Recent studies disclosed the potential for composite reinforcement using a lesser known NLF: guaruman fiber [21,22,23]. This fiber is extracted from the stem of a plant botanically named *Ischinosiphon körn*, which is naturally grown mainly in the Amazon region [24]. Preliminary morphological and mechanical characterization revealed a potential for composite reinforcement [21,22]. Indeed, ballistic results of epoxy plates with 30 vol% guaruman fibers [23] in cross-laid configuration display superior performance against both 7.62 mm and 0.22 caliber ammunition, compared to other armors—such as those made with epoxy composites reinforced with 30 vol% pineapple leaf fibers (PALF) [16] or 30 vol% sedge fibers [25].

In order to complement the mechanical properties of the investigated guaruman fiber composites intended for engineering applications under dynamic loads, this brief report evaluates the Izod impact strength. Herein is discussed: (1) the effects of different amounts of reinforcement on the impact resistance; (2) the main observed failure mechanism under dynamic loading; and (3) the effectiveness of reinforcement with the guaruman fiber in comparison to other NLFs reinforcing epoxy matrix composites under the same conditions.

## 2. Materials and Methods

### 2.1. Materials

**Figure 1:**
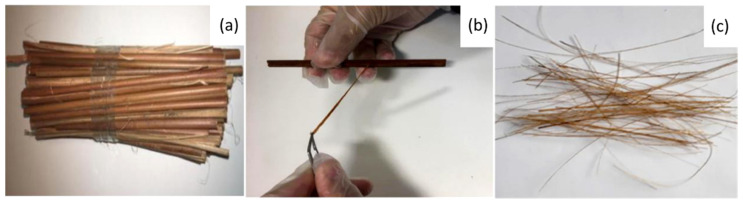
Processing of guaruman fiber: (**a**) stems, (**b**) mechanical separation of fibers, and (**c**) a bundle of guaruman fibers.

As illustrated in Figure 1, stems of the guaruman plant were purchased in a local market in the city of Belem, state of Para, in the north of Brazil. Splints were mechanically cut from each stem, as shown in Figure 1a. Guaruman fibers were carefully separated from each splint with a surgical blade (Figure 1b). Figure 1c presents the guaruman fibers as a bundle prior to their application as reinforcement for polymer matrix composites.

**Figure 2:**
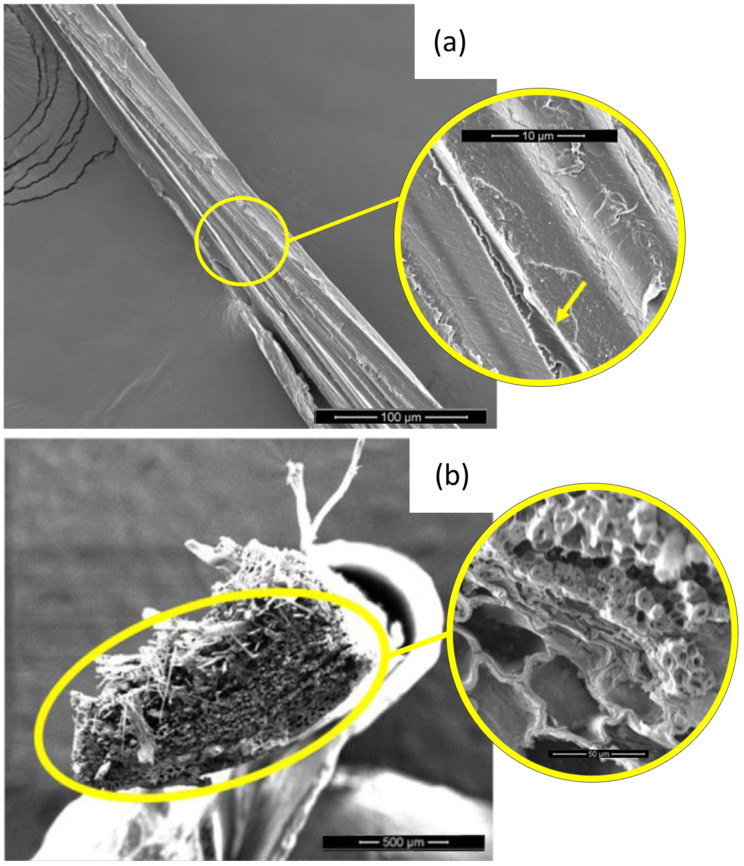
SEM images of a guaruman fiber: (**a**) lateral view with microfibrils, and (**b**) cross-section with large holes and cylindrical microfibrils. Adapted from [22].

Figure 2 shows the scanning electron microscopy (SEM) image of a guaruman fiber with enhanced magnification in both lateral (Figure 2a) and cross-section (Figure 2b) views. In this figure one should note that, like any NLF, the guaruman fiber is composed of microfibrils (Figure 2a) along its entire length. The fiber cross-section in Figure 2b reveals the characteristic lumen as well as large holes and cylindrical microfibrils. The lumen and holes contribute to a lower density, while the microfibrils support the fiber strength [22].

Epoxy resin type diglycidyl ether bisphenol-A (DGEBA) hardened with triethylenetetramine (TETA) catalyst, used in a stoichiometric ratio of 13 parts per 100 (phr) of resin, was applied as a composite matrix. Both DGEBA and TETA were fabricated by Dow Chemical and commercially supplied by Epoxy Fiber, Brazil.

### 2.2. Composite Processing

The as-extracted guaruman fibers (Figure 1c) were cleaned and dried in a stove at 80 °C for 24 h until a constant weight was obtained. Continuous and aligned 150-mm-long fibers were accommodated in layers inside a steel mold, with dimensions of 150 × 120 × 12 mm^3^. Composite plates were prepared by hand lay-up with 10, 20, and 30 vol% of guaruman fibers layers, and then pouring still-fluid DGEBA/TETA epoxy to fill the molds’ empty space. The precise volume fractions of guaruman fibers were determined based on corresponding weighed amounts, which were calculated by the fiber density of 0.57 g/cm^3^ [22]. Upon closing, the mold was kept in a Skay hydraulic press (Sao Paulo, Brazil) under a pressure of 3 MPa at room temperature (RT) for 24 h. After unmolding, the composite plate was post-cured for one week at RT.

### 2.3. Izod Impact Test

The composites’ impact resistance was evaluated via Izod impact test in 20 notched specimens, for each distinct volume fraction of guaruman fiber, cut from corresponding plates in a standard prismatic shape of 60.25 × 12.7 × 10 mm^3^, as per ASTM D256. A V-notch with an angle of 45° and 2.54 mm deep was produced in each specimen using a Pantec Iz/Ch-50 single-tooth carver. The Izod tests were carried out using a model XC-50 Pantec pendulum operating with a 22 J hammer.

### 2.4. Statistical Validation

Weibull analysis in association with analysis of variance (ANOVA) and Tukey’s test were conducted in order to statistically validate the level of reliability and significance of the Izod impact results. The Weibull parameters *β* and *θ* in the frequency distribution function are related as:

(1) $$f\left(x\right)=\exp\left[{\left(\frac{x}{\theta }\right)}^{\beta }\right]$$

Together with the precision R^2^, these parameters contribute to evaluating the level of data reliability. The ANOVA and Tukey’s test allow us to assume and determine the existence of differences between the average and standard deviation values of absorbed impact energy, with a 95% level of confidence.

### 2.5. Scanning Electron Microscopy (SEM)

SEM images of Izod impact-ruptured composite specimens were analyzed with a Quanta FEG 250 Fei microscope operating with secondary electrons at 10 KV. Samples were gold-sputtered before SEM observation.

## 3. Results

### 3.1. Impact Resistance

**Figure 3:**
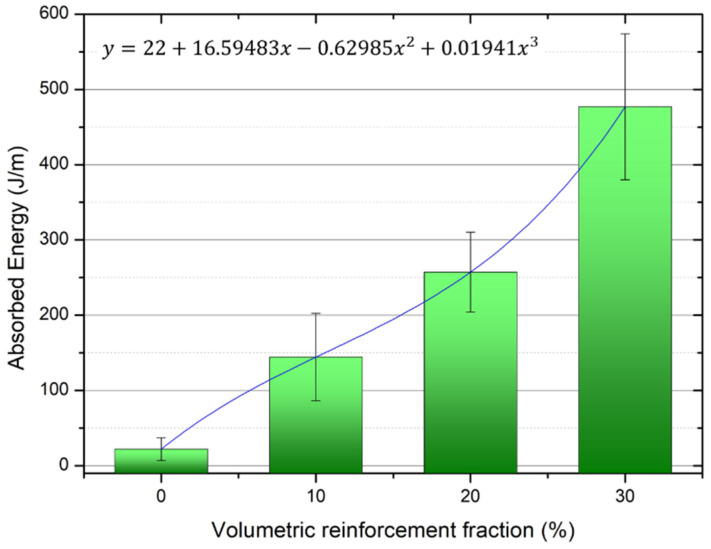
Variation of Izod absorbed impact energy with the volume fraction of guaruman fiber in the epoxy matrix composites. Neat epoxy value obtained from [26].

Figure 3 shows the variation in the absorbed energy, associated with the impact resistance, as a function of the volume fraction of guaruman fiber incorporated into the epoxy composite. In this figure, one may notice a significant increase in the impact energy absorption; such an increment of resistance may be approximated by a third-degree polynomial function, as noted. Another point worth mentioning is that an energy absorption 20 times higher than those measured for the neat epoxy condition [22] was obtained for the 30 vol% guaruman fiber composites.

### 3.2. Statistical Validation

**Figure 4:**
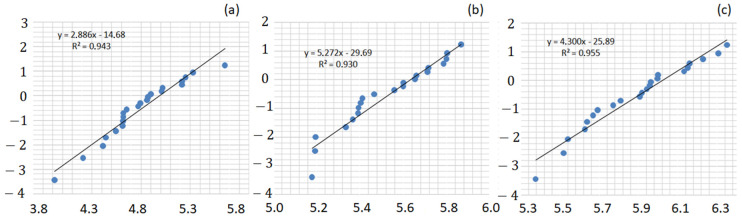
Weibull frequency distribution graph for the epoxy composites with different volume fractions of guaruman fiber: (**a**) 10, (**b**) 20, and (**c**) 30 vol%.

Figure 4 shows the Weibull graphs for the guaruman fiber composites. In principle, the reasonable straight-line adjustment of corresponding data points might be related to the same impact resistance mechanism for each distinct composite.

Table 1 presents the Weibull parameters for the guaruman fiber composites. The relatively high standard deviation could imply a possible statistical similarity between composites with 20 and 30 vol% of guaruman fiber. Nevertheless, the other two statistical analyses used in this investigation—ANOVA, and Tukey’s test—reject this possibility, as will be further discussed. This may be also associated with the different fracture mechanisms found to be operating for each composite, as will be further discussed in the SEM analysis. Another point worth mentioning regarding the Weibull results in Table 1 is the satisfactory statistical precision (R^2^ > 0.9) associated with the impact resistance results for each investigated composite.

Table 2 presents the ANOVA for the impact resistance results of guaruman fiber composites. The data in this table indicate that the hypothesis of equality between the Izod absorbed energy values (Figure 3 and Table 1) is rejected with a 95% level of confidence, because Fcalc = 41.03 is much higher than Fcritical (tabulated) = 3.18. Therefore, the impact resistance associated with each investigated volume fraction of guaruman fiber is statistically different than the others.

In order to verify which volume fraction of guaruman fiber displays the best impact resistance, Tukey’s test was applied to compare individual performance with a 95% level of confidence. Table 3 presents the honestly significant difference (HSD) obtained via Tukey’s test. The calculated HSD was found to be 61.41 J/m, and differences above this value are considered significant. The values in Table 3 reveal that the impact resistance of the 30 vol% guaruman fiber composite is indeed superior to that of the other composites.

### 3.3. Scanning Electron Microscopy Analysis

**Figure 5:**
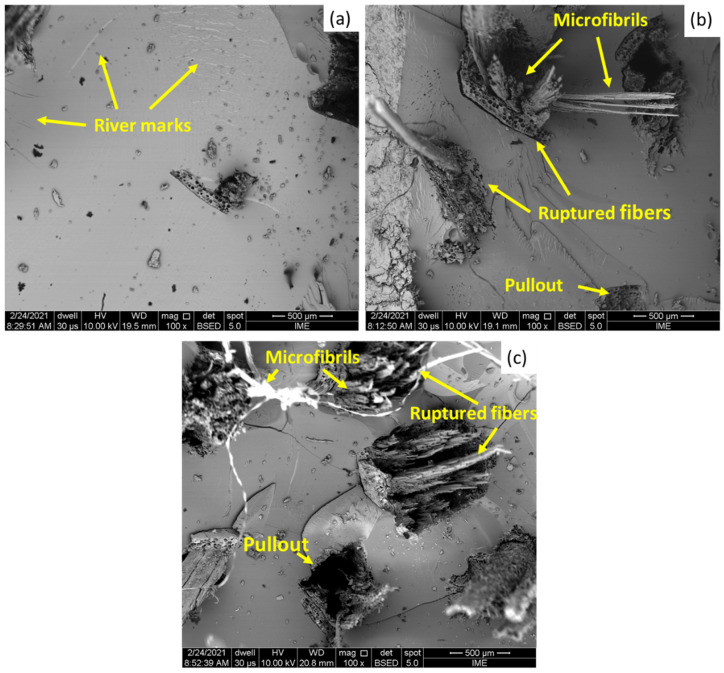
SEM images of the fractured surfaces of the epoxy composites with different volume fractions of guaruman fiber: (**a**) 10, (**b**) 20, and (**c**) 30 vol%.

Figure 5a–c presents the SEM analysis of the condition of the fractured surfaces of the investigated composites.

One may notice in Figure 5a the brittle characteristics exhibited by the composite with the lowest amount of fiber reinforcement (10 vol% of guaruman fiber), which may be associated with the ineffective degree of reinforcement. In this case, the brittleness of the epoxy matrix can be held responsible as the main mechanism of failure of this composite. This behavior is evidenced by the appearance of river marks in the epoxy matrix. Similar behavior was reported by Garcia Filho et al. [18] for an epoxy matrix composite reinforced with piassava fibers under high-strain-rate conditions, especially for composites with a lower volume percentage of fiber reinforcement.

The brittleness of the polymeric matrix failure mechanism was observed in all composites investigated, and was also associated with others failure modes involving the influence of the fibers. Increasing the amount of guaruman fiber as reinforcement for the composite (20 and 30 vol% composites) increased the impact resistance of the material, as shown in Figure 3. Therefore, one may expect that more complex fracture mechanisms would be activated, including the fracture of guaruman fibers as well as fiber pull-out, as noted in Figure 5b,c.

Regarding the individual guaruman fibers in Figure 5b,c, their rupture occurred in association with broken microfibrils that contribute additional free surface, increasing the absorbed impact energy.

It is proposed that these are the main mechanisms that contribute to the dissipation of the Izod impact energy of guaruman fiber composites. The fracture of the fibers is facilitated by the rupture of the internal channels that are associated with the cellular structure (Figure 2b) inherent to the microstructure of NLFs. This cellular structure consists of an arrangement of several holes and microfibrils with some mechanical and functional role [7].

**Figure 6:**
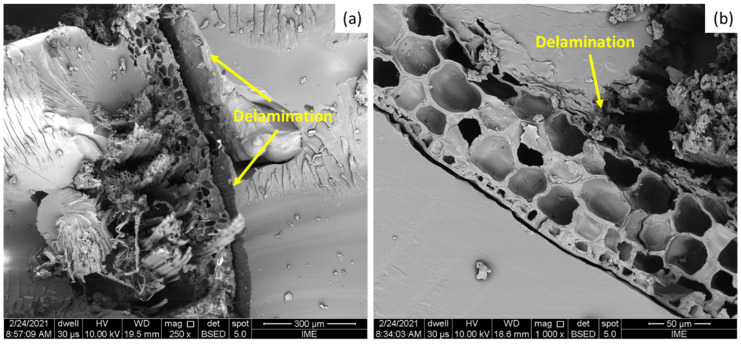
SEM images of the fractured surfaces of the epoxy composites reveal: (**a**) delamination; and (**b**) the morphology of the guaruman fiber, exhibiting several voids.

Finally, in Figure 5c one may clearly verify a high amount of fiber reinforcement in comparison to the 10 and 20 vol% reinforcement (Figure 5a,b respectively). This then implies a higher effectiveness of the fiber reinforcement. As cracks are formed throughout the matrix, their paths are blocked and stopped by the fibers. Pullout mechanisms, as shown in Figure 6a,b, are characterized by the withdrawal of the fiber from the polymeric matrix, and commonly used to describe failure in composites reinforced with fibers with low interfacial adhesion between fiber and matrix.

The low interfacial adhesion is responsible for triggering the pull-out effect in this kind of composite, and can also be the source of another important failure mechanism for epoxy composites reinforced with NLFs—delamination. This behavior might be associated with the different natures of the polymer matrix and the natural fiber itself. While the polymer matrix tends to display hydrophobic characteristics, the guaruman fiber—like most NLFs—exhibits a hydrophilic nature. This difference impairs the interfacial adhesion of the reinforcement in the matrix; thus, cracks tend to occur between these interfaces, and delamination takes place, as shown in Figure 6a.

Another noteworthy point can be observed in Figure 6; in this figure, one may observe the delamination between fiber and matrix, as observed in Figure 6a. However, it is also important to note the high number of voids and pores—as shown with high magnification in Figure 6b—that are inherent to the guaruman fiber. Such high pore content can be directly associated with the low density calculated for this fiber [22]. This could be considered a remarkable advantage of the use of the guaruman fiber in comparison to many other NLFs as reinforcement for polymer matrix composites, as will be further discussed.

### 3.4. Comparison to Other NLFs Reinforcing Epoxy Composites

Table 4 presents a comparative list of Izod absorbed energy obtained in notched specimens of different 30 vol% natural-fiber-reinforced DGEBA/TETA epoxy composites.

The overall reinforcement effect caused by 30 vol% guaruman fiber in the impact resistance of epoxy composites is one of the highest when compared to other impact-tested natural fiber epoxy composites under similar conditions.

Guaruman, mallow [29], jute [30], and ramie [5] exhibit the highest Izod impact resistance among all investigated NLFs reinforcing epoxy composites. The values observed for these four NLFs’ reinforcement are above 425 J/m; however, the ramie fiber [5] stands out, with the best energy absorption under Izod impact conditions. It is important to notice that when the specific impact resistance is considered—i.e., the impact resistance divided by the density of the composite—the superior behavior of guaruman/epoxy composites stands out.

Considering the specific resistance, it would be possible to achieve values around 507 J/m per g/cm^3^ for the guaruman reinforcement against 465 J/m per g/cm^3^ for the ramie reinforcement under the same conditions. The guaruman fibers display one of the lowest densities of NLFs ever reported. Only the sedge [25] and fique [27] fibers are comparable to the guaruman fiber, presenting densities around 0.5 g/cm^3^. The low density, which may be attributed to the high amount of porosity observed (as shown in Figure 2b and Figure 6b), maximizes this specific impact property. In fact, this could favor the application of guaruman fiber composites where high impact energy absorption associated with low weight is required, as is the case for of bulletproof vests, as well as car and aerospace components.

## 4. Conclusions

The Izod impact resistance of epoxy composites reinforced with a relatively unknown guaruman fiber from the Amazon region was reported to complement previously published mechanical properties.

- The incremental increase in the volume fraction of fiber reinforcement resulted in an increase in the impact resistance of the composite. Composites reinforced with 30 vol% of guaruman fiber exhibited an improvement of over 20 times in terms of impact energy absorption in comparison to a neat epoxy condition. The measured results were validated with 95% confidence by Weibull analysis, ANOVA, and Tukey’s statistical test.
- A shift was observed in the main failure mechanisms of the composites’ energy absorption. The composite with the lowest amount of reinforcement (10 vol%) exhibited brittle characteristics, while higher amounts of reinforcement (20 and 30 vol%) resulted in a combination of complex mechanisms, such as fracture of fibers, pullout, and delamination, which are proposed to be directly associated with the high impact energy absorption by these composites.
- The results obtained with 30 vol% reinforcement with guaruman fiber were compared with other NLFs reinforcing epoxy matrix composites. The reinforcement guaranteed by the guaruman fiber was one of the highest ever reported for NLF composites. The low density exhibited by the guaruman fiber directly impacts on the specific properties of this kind of composite. This combination of high impact resistance and low density is desirable in many applications, such as ballistics, as well as in the vehicular and aerospace industries.

## Figures and Tables

**Table 1:** Weibull parameters for the impact resistance of guaruman fiber epoxy composites.

Epoxy Composite	10 vol% Guaruman	20 vol% Guaruman	30 vol% Guaruman
**E_absorbed_ (J/m)**	144.37	257.21	375.01
**Standard deviation**	57.96	53.09	96.96
***β***	2.89	5.27	4.30
***θ***	161.87	279.12	411.73
**R^2^**	0.95	0.93	0.96

**Table 2:** ANOVA for the Izod absorbed impact energy of epoxy composites with different volume fractions of guaruman fibers.

Variation Causes	DF	SS	MS	Fcalc	Fcritical (tab.)
**Treatment**	2	481,813.59	240,906.80	41.03	3.18
**Residue**	51	299,463.80	5871.84		
**Total**	53	781,277.39			

SS: sum of squares; MS: mean square; DF: degree of freedom.

**Table 3:** HSD as measured by Tukey’s test for the Izod absorbed impact energy of epoxy composites with different volume fractions of guaruman fibers.

Epoxy Composites with GF Reinforcement	GF10 vol%	GF20 vol%	GF30 vol%
**GF10 vol%**	0.00	114.45	231.37
**GF20 vol%**	114.45	0.00	116.92
**Gf30 vol%**	231.37	116.92	0.00

**Table 4:** Izod absorbed impact energy of 30 vol% natural fibers reinforcing epoxy composites with notched specimens.

Fiber Reinforcement	Average Absorbed Energy (J/m)	Fiber Density (g/cm^3^)	Specific Absorbed Energy (J/m per g/cm^3^)	Reference
Guaruman (Ischinosiphon körn)	477	0.57	506.91	PW
Ramie (Boehmeria nivea)	567	1.5	464.75	[5]
Sedge (Cyperus malaccensis)	63	0.46	69.38	[25]
Carnauba (Copernicia prunifera)	137	1.34	116.89	[26]
Fique (Furcraea andina)	134	0.67	138.00	[27]
Tucum (Astrocaryum vulgare)	166	1.61	132.48	[28]
Mallow (Urena lobata)	499	1.00	466.35	[29]
Jute (Corchorus capsularis)	426	1.44	354.41	[30]
Hemp (Cannabis sativa)	134	1.52	109.29	[31]

## Data Availability

Not applicable.

## References

[1] Bledzki A.K., Gassam J. (1999). Composites reinforced with cellulose based fibers. Prog. Polym. Sci..

[2] Thakur V., Thakur M.K., Gupta R.K. (2014). Review: Raw natural fiber-based polymer composites. Int. J. Polym. Anal. Charact..

[3] Sanjay M., Madhu P., Jawaid M., Senthamaraikannan P., Senthil S., Pradeep S. (2018). Characterization and properties of natural fiber polymer composites: A comprehensive review. J. Clean. Prod..

[4] Garcia Filho F.C., Luz F.S., Oliveira M.S., Pereira A.C., Costa U.O., Monteiro S.N. (2020). Thermal behavior of graphene oxide-coated piassava fiber and their epoxy composites. J. Mater. Res. Technol..

[5] Garcia Filho F.C., Luz F.S., Nascimento L.F.C., Satyanarayana K.G., Drelich J.W., Monteiro S.N. (2020). Mechanical Properties of Boehmeria nivea natural fabric reinforced epoxy matrix composite prepared by vacuum-assisted resin infusion molding. Polymers.

[6] Zhang Z., Cai S., Li Y., Wang Z., Long Y., Yu T., Shen Y. (2020). High performance of plant fiber reinforced composites–A new insight from hierarchical microstructures. Compos. Sci. Technol..

[7] Kalia S., Kaith B.S., Kaur I. (2011). Cellulose Fibers: Bio- and Nano-Polymer Composites.

[8] Luz F.S., Garcia Filho F.C., del-Rio M.T.G., Nascimento L.F.C., Pinheiro W.A., Monteiro S.N. (2020). Graphene-incorporated natural fiber polymer composites: A first overview. Polymers.

[9] Dunne R., Desai D., Sadiku R., Jayaramudu J. (2016). A review of natural fibres, their sustainability and automotive applications. J. Reinf. Plast. Compos..

[10] Potluri R., Krishna N.C. (2020). Potential and Applications of Green Composites in Industrial Space. Mater. Today Proc..

[11] Mansor M.R., Nurfaizey A.H., Tamaldin N., Nordin M.N.A. (2019). Natural fiber polymer composites: Utilization in aerospace engineering. Biomass Biopolym. Based Mater. Bioenergy.

[12] Youssef A.M., El-Sayed M. (2018). Bionanocomposites materials for food packing applications: Concepts and future outlook. Carbohydr. Polym..

[13] Krishna N.K., Prasanth M., Gowtham R., Karthic S., Madhavan M.K. (2018). Enhancement of properties of concrete using natural fibers. Mater. Today Proc..

[14] Barbosa J.D.V., Azevedo J.B., Silva M.C.P., Filho F.C.G., del-Rio T.G. (2020). Development and characterization of WPCs produced with high amount of wood residue. J. Mater. Res. Technol..

[15] Silva G., Kim S., Aguilar R., Nakamatsu J. (2020). Natural fibers reinforcement additives for geopolymers—A review of potential eco-friendly applications to the construction industry. Sustain. Mater. Technol..

[16] Luz F.S., Garcia Filho F.C., Oliveira M.S., Nascimento L.F.C., Monteiro S.N. (2020). Composites with natural fibers and conventional materials Applied in a hard armor: A comparison. Polymers.

[17] Pereira A.C., Assis F.S., Garcia Filho F.C., Oliveira M.S., Lima E.S., Lopera H.A.C., Monteiro S.N. (2019). Evaluation of the projectile’s loss of energy in polyester composite reinforced with fique fiber and fabric. Mater. Res..

[18] Garcia Filho F.C., Oliveira M.S., Pereira A.C., Nascimento L.F.C., Matheus J.R.G., Monteiro S.N. (2020). Ballistic behavior of epoxy matrix composites reinforced with piassava fiber against high energy ammunition. J. Mater. Res. Technol..

[19] Nurazzi N.M., Asyraf M.R.M., Khalina A., Abdullah N., Aisyah H.A., Rafiqah S.A., Sabaruddin F.A., Kamarudin S.H., Norrrahim M.N.F., Ilyas R.A. (2021). A Review on Natural Fiber Reinforced Polymer Composite for Bullet Proof and Ballistic Applications. Polymers.

[20] Nayak S.Y., Sultan M.T.H., Shenoy S.B., Kini C.R., Samant R., Shah A.U.M., Amuthakkannan P. (2020). Potential of natural fibers in composites for ballistic applications—A review. J. Nat. Fibers.

[21] Pinheiro M.A., Gomes L.G., Silva A.C.R., Candido V.S., Reis R.H.M., Monteiro S.N. (2019). Guaruman: A natural Amazonian fiber with potential for Polymer composite reinforcement. Mater. Res..

[22] Reis R.H.M., Nunes L.F., Oliveira M.S., Veiga Junior V.F., Garcia Filho F.C., Pinheiro M.A., Silva A.C.R., Candido V.S., Monteiro S.N. (2020). Guaruman fiber: Another possible reinforcement in composites. J. Mater. Res. Technol..

[23] Reis R.H.M., Nunes L.F., Luz F.S., Candido V.S., Silva A.C.R., Monteiro S.N. (2021). Ballistic performance of guaruman fiber composites in multilayered armor system and single target. Polymers.

[24] Global Biodiversity Information Facility. https://www.gbif.org/pt/.

[25] Neuba L.M., Junio R.F.P., Ribeiro M.P., Souza A.T., Lima E.S., Garcia Filho F.C., Figueiredo A.B.H.S., Braga F.O., Azevedo A.R.G., Monteiro S.N. (2020). Promising mechanical, thermal, and ballistic properties of novel epoxy composites reinforced with Cyperus malaccesis sedge fiber. Polymers.

[26] Junio R.F.P., Nascimento L.F.C., Neuba L.M., Souza A.T., Moura J.V.B., Garcia Filho F.C., Monteiro S.N. (2020). Copernicia prunifera leaf fiber: A promising new reinforcement for epoxy composites. Polymers.

[27] Oliveira M.S., Garcia Filho F.C., Luz F.S., Pereira A.C., Demosthenes L.C.C., Nascimento L.F.C., Lopera H.A.C., Monteiro S.N. (2019). Statistical analysis of notch toughness of epoxy matrix composites reinforced with fique fabric. J. Mater. Res. Technol..

[28] Oliveira M.S., Luz F.S., Souza A.T., Demosthenes L.C.C., Pereira A.C., Garcia Filho F.C., Braga F.O., Figueiredo A.B.H.S., Monteiro S.N. (2020). Tucum fiber from Amazon Astrocaryum vulgare palm tree: Novel reinforcement for polymer composites. Polymers.

[29] Costa U.O., Nascimento L.F.C., Garcia J.M., Bezerra W.B.A., Monteiro S.N. (2020). Evaluation of Izod impact and bend properties of epoxy composites reinforced with mallow fibers. J. Mater. Res. Technol..

[30] Mishra V., Biswas S. (2013). Physical and Mechanical Properties of Bi-directional Jute Fiber Epoxy Composites. Procedia Eng..

[31] Ribeiro M.P., Neuba L.M., Silveira P.H.P.M., Luz F.S., Figueiredo A.B.H.S., Monteiro S.N., Moreira M.O. (2021). Mechanical, termal and ballistic performance of epoxy composites reinforced with Cannabis sativa hemp fabric. J. Mater. Res. Technol..

